# Pathogenetic Involvement of Autophagy and Mitophagy in Primary Progressive Multiple Sclerosis

**DOI:** 10.1111/jcmm.70455

**Published:** 2025-04-21

**Authors:** Simone Patergnani, Michele Laudisi, Massimo Bonora, Giulio Righes, Sofia Straudi, Mariusz R. Wieckowski, Ilaria Casetta, Luana Semenzato, Konstantinos Koutsikos, Veronica Zanato, Carlotta Giorgi, Paolo Pinton

**Affiliations:** ^1^ Department of Medical Sciences, Section of Experimental Medicine, and Laboratory for Technologies of Advanced Therapies (LTTA) University of Ferrara Ferrara Italy; ^2^ Department of Neuroscience “S. Anna” University Hospital Ferrara Italy; ^3^ Laboratory of Mitochondrial Biology and Metabolism Nencki Institute of Experimental Biology of the Polish Academy of Sciences Warsaw Poland; ^4^ IRCCS San Camillo Hospital Venice Italy; ^5^ Biomedical Research Center Kansai Medical University Osaka Japan

**Keywords:** ATG5, ATG7, biomarker, GFAP, lactate, Optineurin, Parkin, serum

## Abstract

Primary progressive multiple sclerosis (PPMS) affects a subset of MS patients and is characterised by continuous progression from the onset. The molecular mechanisms underlying PPMS are poorly understood, and therapeutic options are limited, with no specific markers for early detection and monitoring. This study investigated the roles of autophagy and mitophagy in PPMS. We found that autophagy markers (ATG5 and ATG7) and mitophagy markers (Parkin and Optineurin) were significantly reduced in the serum of PPMS patients compared to control and relapsing‐remitting MS (RRMS) individuals. This reduction was associated with an increase in markers indicative of neurodegeneration and mitochondrial dysfunction. Additionally, a positive correlation between autophagy and mitophagy proteins in the PPMS group suggests that these mechanisms are reciprocally associated and modulated in PPMS. Our investigation reveals that autophagy and mitophagy are actively involved in PPMS and exhibit distinct patterns across MS subtypes. Measurements of circulating components related to autophagy and mitophagy could serve as potential biomarkers for early PPMS detection.

**Abbreviations**
ATG: autophagy related
DMTs: disease‐modifying therapies
GFAP: Glial Fibrillary Acidic Protein
MS: multiple sclerosis
OPTN: Optineurin
PPMS: primary progressive multiple sclerosis
RRMS: relapsing remitting multiple sclerosis
SPMS: secondary progressive multiple sclerosis

## 1 Introduction

Multiple sclerosis (MS) is one of the most common major diseases of the central nervous system and represents the leading cause of disability in young adults. About 90% of people with MS are diagnosed with the relapsing‐remitting multiple sclerosis (RRMS) type. During RRMS, a person with MS experiences periods of relapses or exacerbation, in which symptoms worsen, followed by periods of partial or complete recovery [1]. Over time, symptoms in RRMS patients may gradually worsen, leading to a transition to the secondary progressive phase (SPMS), characterised by the steady worsening of neurologic function and a consequent accumulation of disability [1]. About 10% of MS patients have the primary progressive MS (PPMS) type, in which the neurological disability gradually increases from the onset [1]. In recent decades, the field of MS has reached significant advancements, leading to the development of novel pharmacological interventions that can attenuate progressive neuronal degeneration and ameliorate clinical symptoms [2]. However, despite these disease‐modifying therapies (DMTs) being effective in reducing the transition from RRMS to SPMS, they have proven to be less effective against the PPMS form, which remains a considerable challenge [3, 4, 5, 6]. This is mainly due to limited knowledge of what exactly drives PPMS and which molecular mechanisms are involved in its onset and progression. Additionally, to date, there are no validated biomarkers for the diagnosis or the prognosis of PPMS [7, 8]. Interestingly, it has been demonstrated that autophagy and mitophagy (two catabolic cellular pathways activated in response to stress conditions and responsible for the cell death and survival) play determinant roles in MS progression. On one hand, autophagy routes can help preserve cellular homeostasis and prevent the neurodegenerative process typical of MS [9, 10, 11, 12]. However, on the other hand, most investigations demonstrate that these processes are highly associated with MS and are responsible for exacerbating disease progression. Indeed, excessive activation of autophagy and mitophagy has been found in vitro and in ex vivo MS models and has been correlated with demyelination [13]. Similar observations were also found in in vivo models of demyelination, where an increase in autophagy pathways correlated with the progressive loss of myelin markers [13, 14]. Consistently counteracting these pathways has been shown to block demyelination and reactivate myelination rates, as well as improve motor performance in animals exposed to demyelinating agents [13]. Further confirming the harmful role of autophagy and mitophagy processes in MS, increased circulating elements related to these pathways were found in sera and cerebrospinal fluid obtained from MS patients and correlated with the active phase of the pathology [15, 16, 17, 18]. Regrettably, all these investigations have focused solely on the RR form of the disease. No research has been conducted to determine whether autophagy and mitophagy also play an active role in PPMS. To address this question, we aimed to investigate the frequency of specific autophagy and mitophagy markers in MS patients with PPMS and compare these levels with those detected in patients with the RRMS form and in control individuals.

## 2 Patients and Methods

### 2.1 Participants and Sampling

Forty‐one PPMS‐affected persons, 41 RRMS‐affected persons and 41 healthy individuals were included in the study. Table 1 provides an overview of the demographic features and clinical findings of participants (Table 1). MS patients were diagnosed based on standard criteria using biochemical and MRI results evaluated by experienced MS neurologists. All samples were taken, stored and analysed under uniform conditions. Serum samples were obtained by centrifuging blood at 1000 *g* at 20°C for 15 min. The supernatants were collected, under sterile conditions, coded, frozen and stored at −80°C in 250 μL aliquots until analysis.

**TABLE 1:** Demographic and clinical features of participating subjects of the study.

	PPMS (*n* = 41)	RRMS (*N* = 41)	Control (*N* = 41)
Female, *n* (%)	27 (70)	29 (70.7)	27 (70)
Age, mean (SD)	55 (10.2)	40 (8.8)	50 (15.01)
Month since first diagnosis, Median, IQR	65 (25.5–120.5)	94 (64.5–167)	
EDSS, Median, IQR	6.5 (5.5–6.5)	2.5 (1.5–3.5)	
DMT			
None	25 (61)	2 (4.9)	
Glatiramer acetate		1 (2.4)	
Interferons	1 (2.4)	4 (9.8)	
Dimethyl fumarate		4 (9.8)	
Teriflunomide		2 (4.9)	
Fingolimod	2 (4.9)	5 (12.2)	
Natalizumab	0	15 (36.6)	
Rituximab	0	0	
Azathioprine	1 (2.4)	0	
Cyclophosphamide	1 (2.4)	0	
Mitoxantrone		0	
Ocrelizumab	11 (26.8)	8 7 (17.1)	
Alemtuzumab		1 (2.4)	

### 2.2 Evaluation of ATG5, ATG7, Optineurin (OPTN), Parkin and GFAP in Serum Samples

Serum concentrations of ATG5, ATG7, OPTN and Parkin were determined by using commercially available enzyme‐linked immunosorbent assay kits (My BioSource, San Diego, CA, USA; MS7209535 for ATG5, MBS732278 for Parkin, MBS062423 for ATG7 and MBS069530 for OPTN; Thermo Fisher Scientific, EEL079 for GFAP) following the manufacturer's instructions, as previously published [13, 17, 19].

### 2.3 Evaluation of Lactate in Serum Samples

Lactate levels were measured using a colorimetric L‐Lactate Assay Kit (Abcam, ab65331) following the manufacturer's instructions as previously published [13, 17, 19]. Briefly, 10 μL of serum was mixed with 40 μL of Lactate Assay Buffer in 96‐well plates, along with a reaction mix containing Lactate Assay Buffer, Substrate Mix and Enzyme Mix. After 30 min of incubation, OD at 450 nm was recorded using a microplate reader.

### 2.4 Statistical Analysis

Statistical analysis was performed by using GraphPad Prism. Normal distribution was assessed using the D'Agostino‐Pearson and the Kolmogorov–Smirnov tests. Since several variables did not meet the normality assumption, multiple comparisons were performed with the non‐parametric Kruskal–Wallis test with Dunn's correction for post hoc analysis. The correlation between variables was evaluated using the Spearman rank correlation coefficient. A *p*‐value less than 0.01 was considered statistically significant.

## 3 Results

**FIGURE 1:**
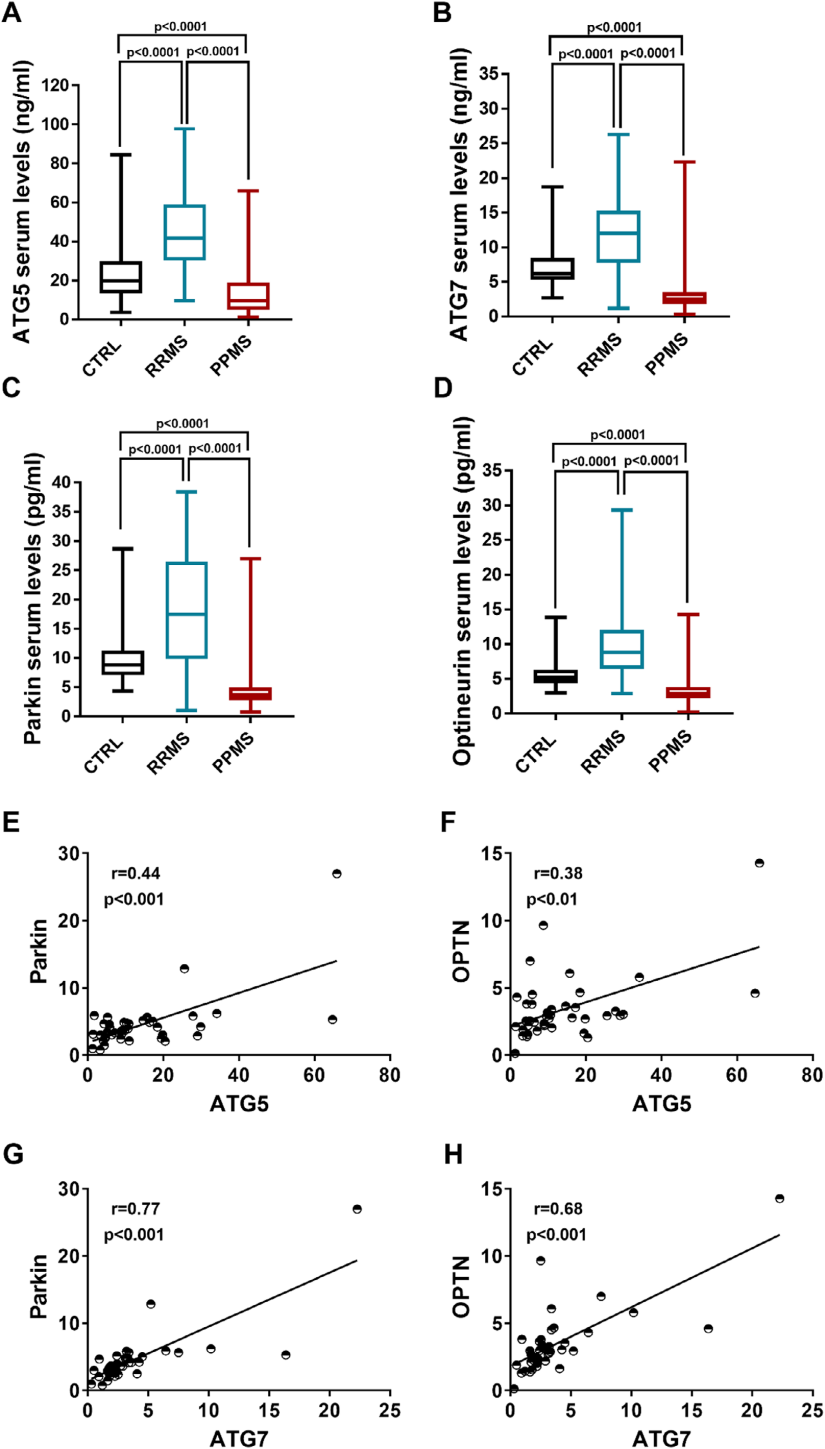
(A–D) Levels of ATG5 (A), ATG7 (B), Parkin (C) and OPTN (D) in the sera of patients with primary progressive multiple sclerosis (PPMS), relapsing remitting multiple sclerosis (RRMS) and healthy individuals (CTRL). The boxes represent the interquartile range (25th–75th percentile). The line within the box denotes the median. The vertical lines extending from the boxes indicate the range of values. (E–H) Spearman's rank correlation analysis between the levels of autophagy markers ATG5 and ATG7 and the mitophagy markers OPTN and Parkin in MS patients affected by PPMS.

Our results demonstrated that the serum level of the autophagy markers ATG5 and ATG7 (Figure 1A,B) and the mitophagy markers OPTN and Parkin (Figure 1C,D) were lower in MS subjects affected by the PPMS form than in those with the RRMS form and in control (CTRL) individuals (Kruskal–Wallis test: *p* < 0.0001). Post hoc analysis revealed: (i) reduced median serum concentrations of ATG5 in PPMS (14.28 ng/mL) compared with CTRLs (25.53 ng/mL) and RRMS (44.47 ng/mL); (ii) reduced median serum concentrations of ATG7 in PPMS (3.68 ng/mL) compared with CTRLs (7.10 ng/mL) and RRMS (11.69 ng/mL); (iii) reduced median serum concentrations of Parkin in PPMS (4.68 pg/mL) compared with CTRLs (9.86 pg/mL) and RRMS (18.37 pg/mL); (iv) reduced median serum concentrations of OPTN in PPMS (3.43 pg/mL) compared with CTRLs (5.62 pg/mL) and RRMS (9.58 pg/mL). Our results corroborated previous investigations [13, 16, 17] which reported increased levels of autophagy and mitophagy elements in the sera of RRMS individuals compared with control subjects (Kruskal–Wallis test: *p* < 0.0001). Additionally, we observed increased neurodegeneration markers in MS samples, with a further escalation in the PPMS form (Figure S1A). PPMS patients also exhibited significantly higher lactate levels (Figure S1B), suggesting impaired mitochondrial function. Consistent with prior research, lactate levels in RRMS patients were comparable to those of controls. Post hoc analysis revealed: (i) increased median serum concentrations of GFAP in PPMS (12.95 pg/mL) compared with CTRLs (0.36 pg/mL) and RRMS (9.24 pg/mL) (Kruskal–Wallis test: *p* < 0.01, *p* < 0.0001); (ii) increased median serum concentrations of lactate in PPMS (1.54 mM) compared with CTRLs (0.72 mM) and RRMS (1.13 mM) individuals (Kruskal–Wallis test: *p* < 0.01, *p* < 0.0001). Our results also described a direct correlation between the levels of the autophagy markers with the mitophagy markers in MS patients affected by the primary progressive form of the disease (*p* < 0.001). Indeed, serum levels of ATG5 positively correlated with Parkin (Figure 1E) and OPTN levels (Figure 1F) (ATG5‐Parkin: Spearman *r* = 0.44, *p* < 0.001; ATG5‐OPTN: Spearman *r* = 0.38, *p* < 0.01). Similarly, a positive correlation was also found by comparing the autophagy element ATG7 with Parkin (Figure 1G) and with OPTN (Figure 1H) (ATG7‐Parkin: Spearman *r* = 0.77, *p* < 0.001; ATG7‐OPTN: Spearman *r* = 0.68, *p* < 0.001).

## 4 Discussion

The novelty of our study is the impressive reduction of autophagy and mitophagy markers in patients with PPMS compared with controls and with MS subjects affected by the RR form. The positive correlations found between the autophagy and mitophagy proteins suggested that both catabolic mechanisms are reciprocally associated and modulated in the serum of PPMS‐affected individuals.

In recent years, considerable improvements in the field of MS demonstrate how autophagy and mitophagy pathways contribute to MS aetiology. Unfortunately, all these investigations were mainly focused on the RRMS form, the major type that plagues MS individuals, and lacked information regarding the involvement of these signalling pathways in the progressive forms of the pathology, the SPMS and PPMS. The SPMS represents the progressive and steady worsening of neurological disability in RRMS. Evidence demonstrates that an early utilisation of DMTs can significantly decrease the transition from the RRMS to SPMS type. Conversely, none of the DMTs used for the other MS forms are effective in PPMS, probably because PPMS has different pathogenetic mechanisms from relapse‐onset MS. However, due to the lack of reliable models reproducing the pathology both in vitro and in vivo, the molecular mechanisms involved in PPMS remain to be elucidated. Our study offers new insights into this knowledge gap. Here, we propose that autophagy and mitophagy mutually cooperate in PPMS. In our previous investigations, we showed that in the symptomatic phase of RRMS, both autophagy and mitophagy processes are particularly activated [16, 17]. Therefore, it was easy to speculate that a comparable or more potent increase could also occur during PPMS. Conversely, our results show that the levels of markers of autophagy and mitophagy are reduced in PPMS when compared to those measured in RRMS. These unexpected results are of particular interest since they suggest that there exists a different evolution of the autophagy and mitophagy processes between the irreversible accumulation of the disability (the progressive phase of MS) and the relapsing phase and therefore during the active state of the pathology.

Furthermore, we demonstrate that the levels of catabolic markers detected in PPMS are also lower than those found in healthy donors, thereby suggesting that an active monitoring of the serum levels of these molecules can help in the identification of the PP form of MS.

Other important findings emerge from our study. Alterations in the mitophagy process indicate a persistent disruption of mitochondrial function, which leads to impaired cellular metabolism. This, in turn, may significantly affect the proper functioning of cells and tissues, especially those that rely on high‐energy demands, such as neurons [13, 20]. A key indicator of this impairment is the shift from oxidative metabolism to glycolysis, resulting in an excessive accumulation of lactate. The physiological production of lactate serves as an important substrate for neuronal cells, being actively utilised by astrocytes and neurons to support energy needs and impulse transmission, as well as by oligodendrocytes for growth and myelination [21, 22]. However, elevated lactate levels can be highly detrimental to nervous cells, leading to intracellular acidification, ATP depletion, oxidative stress, impaired oligodendrocyte maturation, and ultimately, cell death [23]. Consistent with other studies [24, 25], our cohort of PPMS patients exhibited elevated lactate levels, suggesting significant mitochondrial dysfunction during the progressive phase of the disease. This hypothesis is further supported by the finding that lactate levels in RRMS patients are comparable to those in healthy controls. Previous studies have attributed this lack of difference to lactate changes occurring exclusively during the active phase of RRMS, while no significant alterations are observed when analysing entire cohorts that include both active and inactive patients. Notably, our analysis revealed that PPMS patients, which tend to have increased neuronal defects [26, 27, 28], exhibit higher levels of neurodegeneration compared to RRMS patients.

The results obtained in our study could also have therapeutic importance. Having found reduced levels in the activity of autophagy and mitophagy machinery in only PPMS patients, it is plausible that treatments improving these cellular processes may produce beneficial effects in the restricted group of patients affected with the PPMS. At the same time, it is important to point out that reductions in autophagy and mitophagy may also be indicative of an uncontrolled activation of autophagy and mitophagy, which trigger larger degradation of autophagy vesicles and, therefore, of autophagy and mitophagy markers. If this latter aspect should be confirmed, a reasonable therapeutic approach to use is one composed of strong autophagy inhibitors and not with enhancers of the autophagy machinery. Further analyses conducted in primary human samples obtained from PPMS are needed to unveil whether the reduction of autophagy and mitophagy observed in serum of PPMS reflects a loss of function or an exacerbation of these catabolic molecular processes. In conclusion, our study demonstrates that autophagy and mitophagy processes play a crucial role in the pathogenesis of PPMS. It emphasises that in this poorly understood form of MS, the levels of circulating elements related to these catabolic processes are lower compared to RRMS and healthy individuals. These findings suggest the potential use of autophagy and mitophagy factors as biomarkers to monitor the progression and different forms of MS and propose new avenues for developing innovative therapeutic approaches against PPMS, for which there is currently no specific treatment regimen.

## Author Contributions

**Simone Patergnani:** conceptualization (equal), data curation (equal), formal analysis (equal), funding acquisition (equal), investigation (equal), methodology (equal), project administration (equal), writing – original draft (equal), writing – review and editing (equal). **Michele Laudisi:** conceptualization (equal), data curation (equal), investigation (equal). **Massimo Bonora:** investigation (equal), writing – review and editing (equal). **Giulio Righes:** investigation (equal), writing – review and editing (equal). **Sofia Straudi:** data curation (equal), investigation (equal), methodology (equal). **Mariusz R. Wieckowski:** writing – review and editing (equal). **Ilaria Casetta:** writing – review and editing (equal). **Luana Semenzato:** writing – review and editing (equal). **Konstantinos Koutsikos:** writing – review and editing (equal). **Veronica Zanato:** writing – review and editing (equal). **Carlotta Giorgi:** conceptualization (equal), writing – review and editing (equal). **Paolo Pinton:** conceptualization (equal), writing – review and editing (equal).

## Disclosure

Permission to reproduce material from other sources: The authors have nothing to report.

## Ethics Statement

The study was approved by the Committee for Medical Ethics in Research of Ferrara (19 January 2017—Study number: 160795) and written informed consent was obtained from all subjects.

## Conflicts of Interest

The authors declare no conflicts of interest.

## Supporting information


Data S1:

## Data Availability

The datasets used and/or analysed during the current study are available from the corresponding author on reasonable request.
