# Supplementary material for: Pathogenetic Involvement of Autophagy and Mitophagy in Primary Progressive Multiple Sclerosis
Source: J Cell Mol Med. 2025 Apr 21;29(8):e70455. doi: 10.1111/jcmm.70455 (PMC12010764; doi:10.1111/jcmm.70455)
Supplement: Supplementary file 1 — Data S1: [file JCMM-29-e70455-s001.docx]

**SUPPLEMENTARY FIGURES**

**S1A**

**S2A**

**Figure S1:** (A-B) Levels of GFAP (A) and Lactate (B) in the sera of patients with primary progressive multiple sclerosis (PPMS), relapsing remitting multiple sclerosis (RRMS) and healthy individuals (CTRL). The boxes represent the interquartile range (25th–75th percentile). The line within the box denotes the median. The vertical lines extending from the boxes indicate the range of values.
